# Massive Orbital Myiasis Caused by *Sarcophaga argyrostoma* Complicating Eyelid Malignancy

**DOI:** 10.1155/2020/5618924

**Published:** 2020-04-30

**Authors:** Anfisa Ayalon, Veronika Yehezkeli, Yossi Paitan, Krzysztof Szpila, Kosta Y. Mumcuoglu, Elad Moisseiev

**Affiliations:** ^1^Department of Ophthalmology, Meir Medical Center, Kfar Saba, Israel; ^2^Sackler School of Medicine, Tel Aviv University, Tel Aviv, Israel; ^3^Clinical Microbiology and Virology Laboratories, Meir Medical Center, Kfar Saba, Israel; ^4^Department of Ecology and Biogeography, Faculty of Biology and Environmental Protection, Nicolaus Copernicus University, Toruń, Poland; ^5^Parasitology Unit, Department of Microbiology and Molecular Genetics, The Hebrew University-Hadassah Medical School, Jerusalem, Israel

## Abstract

*Purpose*. To report a case of massive orbital myiasis caused by the larvae of *Sarcophaga argyrostoma*, complicating eyelid malignancy. *Observations*. A 98-year-old man first presented to our clinic noted to have a fast-growing lesion on his right upper and lower eyelids. Squamous cell carcinoma of the eyelids was highly suspected, and surgical excision was advised, but the patient refused any surgical or nonsurgical intervention. For the next eight months, the patient's family members continued to observe a high rate of tumor growth accompanied by deterioration of the general condition. During this whole period, the patient rejected admission to the hospital and was observed by nursing home staff. He was admitted to the emergency room in cachexic, unresponsive condition with fetid discharge and multiple live maggots crawling out from a large necrotic mass over the right orbit. On examination, no eyelids, eyeball, or other ocular tissue could be seen, while an extension of necrotic mass to forehead and midcheek was noted. Manual removal of larvae was performed. The patient passed away eight hours after his admission and larval removal. The maggots were identified as the third-instar larvae of *Sarcophaga argyrostoma*. *Conclusions and Importance*. This is the first reported case of home-acquired, massive orbital myiasis by *S*. *argyrostoma*. This case illustrates the crucial role of fly control as part of medical and home care in immobile patients. Moreover, it shows the importance of awareness by nursing home staff, paramedical, and medical personnel of possible myiasis, especially in bed-bound patients with skin malignancies and open wounds.

## 1. Introduction

Myiasis is a condition caused by the infestation of humans or animals by fly larvae of the order Diptera. According to the locality of the affected area, human myiasis is usually classified as cutaneous, oral, ocular, nasal, urogenital, and gastrointestinal. Facultative myiasis is caused by fly larvae which usually use decaying organic material such as feces and corpses as medium to develop and can rarely infest animals and humans, e.g., when they are affected by open infected wounds. Obligatory myiasis agents are able to penetrate also the intact skin and are always parasitic, e.g., the spotted flesh fly (*Wohlfahrtia magnifica*), which is common also in the Mediterranean countries [1]. *Sarcophaga argyrostoma* is a cosmopolitan and synanthropic species and is known to cause facultative myiasis in humans and animals [2]. Nosocomial myiasis is a very infrequent phenomenon but can have legal consequences and economic burden and cause prolonged hospitalization, disability, and even death [3–5]. Exposed skin malignancies and open wounds are risk factors of myiasis, especially in immobile patients [6].

We report a case of home-acquired orbital myiasis caused by larvae of *S*. *argyrostoma*, complicating course of eyelid malignancy.

## 2. Case Report

**Figure 1:**
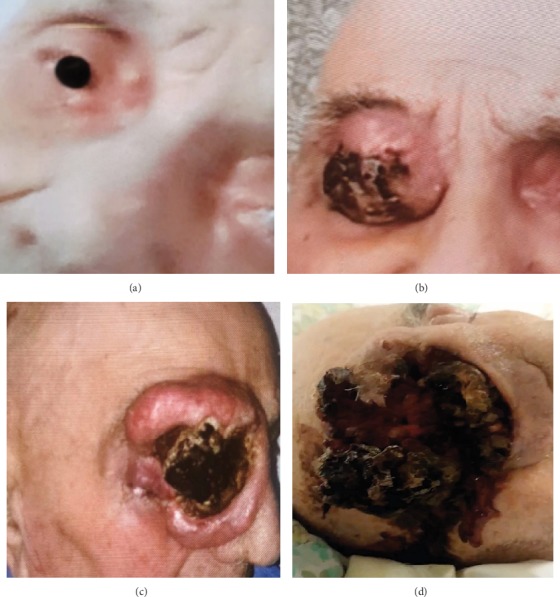
(a) Patient at the first presentation to the oculoplastic clinic. Note nodule-like lesions on the nasal part of his right upper and lower eyelids. (b, c) The high rate of tumor growth. Large, indurated tumor with ulcerated center can be seen. (d) Eight months after the first examination. Large ulcerated squamous cell carcinoma in the right orbital area, note the extension of necrotic mass to forehead and midcheek with multiple maggots.

**Figure 2:**
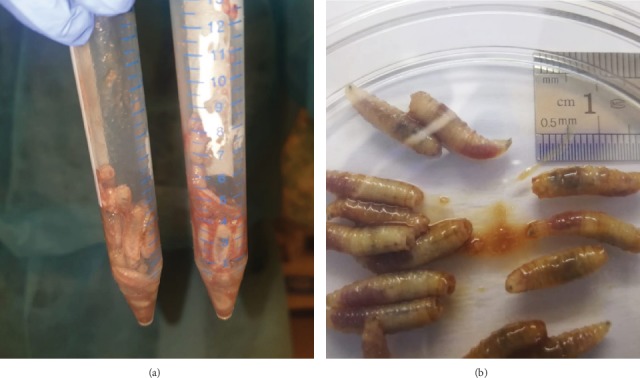
(a, b) Maggots collected after removal from the patient.

**Figure 3:**
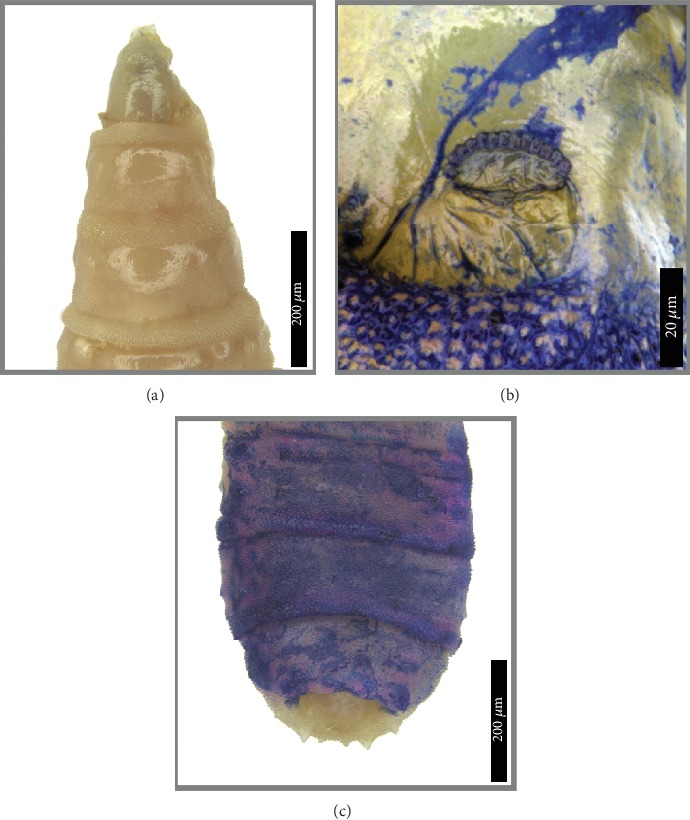
Third-instar larva of *Sarcophaga argyrostoma*. (a) Pseudocephalon and thoracic segments, dorsal view. (b) First thoracic segment, anterior spiracle, contrasted with ink marker. (c) Sixth and seventh abdominal segments and anal division, dorsal view, contrasted with ink marker.

A 98-year-old man with a history of ischemic heart disease, dyslipidemia, and cerebral stroke was first referred to our oculoplastic clinic due to fast-growing lesions in both eyelids from the right side. Squamous cell carcinoma of the eyelids was highly suspected, but the patient refused any further diagnostic or treatment procedures, including biopsy or surgical removal of the lesions (Figure 1(a)). Over the next eight months, the patient refused admission to the hospital and was observed at his home by nursing home staff. The patient's family (members) continued to note a high rate of tumor growth accompanied by deterioration of the general condition (Figures 1(b) and 1(c)). During this period, the patient became unresponsive, cachexic, and had a large orbital tumor mass. When admitted to the emergency room, fetid discharge and multiple live maggots crawling out from a large necrotic over the right orbit could be seen, while he showed a markedly deteriorated general status. On examination, no eyelids, eyeball, or other ocular tissue could be seen on the right side of his face while the necrotic mass extended to forehead and midcheek (Figure 1(d)). Despite intensive treatment, the patient passed away eight hours later. Overall, 32 larvae were removed from the patient and morphologically identified as the third larval instars of *S*. *argyrostoma* [7–9] (Figures 2 and 3).

## 3. Discussion

Squamous cell carcinoma (SCC) is reported as the second most common nonmelanoma skin cancer and the second most common eyelid malignancy [10, 11]. Advanced SCC is challenging to treat and is also a risk factor for unintended infestation with fly larvae. According to the literature, ophthalmomyiasis is a condition that can be seen in all age groups and, depending on the site of larval invasion, can be classified into external, internal, or orbital [6]. Orbital myiasis is a very rare form of this condition and occurs when large numbers of larvae invade the orbit and destroy its contents [12]. Due to medicolegal and sanitary issues, it is very important to record and correctly identify the species involved in myiasis, especially in cases of nosocomial acquired one. Hospital acquired ophthalmomyiasis by *S*. *argyrostoma* was reported only once in the literature [13]. There are previous reports of orbital myiasis, complication course of eyelid tumor [6, 14], but to the best of our knowledge, our report is the first one that describes a case of home-acquired orbital myiasis caused by *S*. *argyrostoma*. The main predisposing factors for the infestation in our patient included a very large area of necrotic tissue offered by the tumor, the patient's poor general condition with multiple medical comorbidities, advanced age, and lack of hygiene and fly control.

Due to lack of standard protocols, management of myiasis can be challenging and should be first focused on its prevention. Awareness by nursing home staff, paramedical, and medical personnel to possible myiasis in immobile patients with skin malignancies and open wounds is crucial. Screens on the windows could prevent flies from entering the rooms were immobilized patients and those with chronic wounds are living or hospitalized, while it is important to eliminate existing flies with insecticides. Proper wound care, hygiene, and adequate nutrition also play an important role in the recovery [14].

Treatment should aim to remove maggots completely, and the simplest measures are irrigation and mechanical removal [10]. Surgical debridement of local site with the use of topical agents, like turpentine oil, chloroform, ethyl chloride, phenol, and olive oil, and systemic treatment with ivermectin were described to be effective in the literature [15, 16]. Although topical and systemic treatments were shown to be effective in the management of myiasis, it should be kept in mind that such insecticides kill larvae in the deep layers of the wound and without removal of neutralized decomposing larvae body intoxication can be initiated. It should be also remembered that third-instar larvae usually stay for additional 1-3 days on the wound and leave the human body to pupate in a dry environment. In the literature, there are few reported cases of orbital myiasis and no previous reports of orbital myiasis caused by *S*. *argyrostoma*. Our patient had an unlikely course and outcome of the eyelid squamous cell carcinoma due to his refusal of any treatment and late presentation. The present case illustrates the importance of prevention and monitoring by medical personnel in immobile and debilitated patients.

## 4. Conclusion

Myiasis is a possible complication of untreated eyelid malignancies. To the best of our knowledge, we reported the first case of massive orbital myiasis caused by *S*. *argyrostoma*. There is a crucial role of fly control as part of medical and home care in immobile patients.

Moreover, our case shows the importance of awareness by nursing home staff, paramedical, and medical personnel of possible myiasis, especially in bad-bound patients with skin malignancies and open wounds.
